# Lupoid Cutaneous Leishmaniasis: A Case Report

**DOI:** 10.14789/ejmj.JMJ25-0056-CR

**Published:** 2026-03-04

**Authors:** HOORIYA SHAFIQ BUTT, AMEERAH KHAN, ALIZA HAMADANI, RABIA SHAUKAT, DANYAL SHAFIQ BUTT, HAROON NABI

**Affiliations:** 1 Department of Dermatology, Ghurki Trust Teaching Hospital, Lahore, Punjab, Pakistan; 1 Department of Dermatology, Ghurki Trust Teaching Hospital, Lahore, Punjab, Pakistan; 2 Department of Internal Medicine, The University of Toledo, Ohio, USA; 2 Department of Internal Medicine, The University of Toledo, Ohio, USA

**Keywords:** cutaneous leishmaniasis, lupus vulgaris, facial plaque

## Abstract

**Background:**

Lupoid Cutaneous Leishmaniasis(LCL) is rare type of cutaneous leishmaniasis that clinically and histologically mimics other granulomatous skin conditions like Lupus Vulgaris. This similarity often cause difficulty in diagnosis, especially in endemic areas.

**Case Presentation:**

We report a case of a 29-year-old male who presented with isolated plaque on right cheek persisting for 2 years. The lesion was slowly progressive, non-tender and had not responded completely to local therapy. Smear of initial lesion revealed +LD bodies but the recurrent lesion showed no amastigotes on smear. Histopathology report demonstrated tuberculoid granuloma.

These findings gave us diagnostic challenge between leishmaniasis and other granulomatous disorders. Based on history, clinical examination and histopathology report our diagnosis was lupoid leishmaniasis. We gave Intramuscular injection of 20mg/kg Meglumine Antimonate (MA) OD for one month. After one month of follow up, we gave Intralesional MA 1-3ml at the base of the lesion 14 times and this resulted in complete cure of patient.

**Discussion:**

This case emphasizes the need of including LCL in the differential diagnosis of chronic granulomatous facial lesions, particularly when there is a history of travel to an endemic location. Early detection and precise diagnosis are critical for avoiding misdiagnosis and unneeded anti-tuberculous treatment.

## Introduction

Cutaneous Leishmaniasis (CL) is an immune mediated disease caused mainly by Leishmania (L.) tropica, L. major, L. mexicana, L. Brasiliense’s, and L. amazonensis^1)^. The disease is prevalent in many countries like Pakistan, Afghanistan, Brazil, and Algeria. 90% of the Leishmania cases were reported in these countries^2)^. It is significant cause of public health concern in these areas^3)^. CL can result in marked disfigurement that leads to humiliation and discrimination^4)^. In case of facial lesion, it is difficult to differentiate it, from Lupus Vulgaris^5, 6)^.

## Case report

29-year-old male resident of Banu, presented with unilateral isolated asymptomatic slowly progressive erythematous plaque on right cheek. The lesion appeared initially as a pea sized papule 2 years ago, gradually enlarging to form a plaque. Smear of this lesion showed positive LD bodies at that time. So, he received 6 Intra-lesional injection of Glucantime 1 and a 1/2 years back after which lesions regressed, leaving behind erythema. However, he noticed reappearance and progressive infiltration of the plaque over the same area in the following months. Cutaneous examination showed solitary, unilateral, ulcerated, erythematous, slightly edematous plaque with well-defined margins on the right side and it was extending 1 cm below the lower eyelid to right malar prominence measuring 3 cm * 4 cm in size. It was non-tender with mild atrophy present in center, with active erythematous margins. Temperature was comparable to normal side and there was no active discharge from the lesion. Nasal, conjunctival and oral mucosa were normal, showing no functional disability. Rest of the skin was normal with no palpable lymph nodes.

A slit skin smear was performed again, which showed no LD bodies. Histological examination showed collection of epithelioid histiocytes forming granulomas surrounded by dense lymphocytic infiltrate. No caseous necrosis was identified. Special stains i.e. PAS and ZN stain were negative. Molecular and immunological tests including PCR for Leishmania, GeneXpert for Mycobacterium tuberculosis, and interferon-gamma release assay (IGRA) were all negative. The patient had no constitutional symptoms suggestive of tuberculosis (such as weight loss, night sweats, or fever). These findings, along with the clinical features and treatment response, supported the diagnosis of Lupoid Cutaneous Leishmaniasis (LCL), acknowledging the known low sensitivity of PCR in this variant. LCL was suspected based on the patient’s residential area and the chronic facial lesion.

Intramuscular injection of 20 mg/kg Meglumine Antimonate OD for one month was given. Laboratory tests and vital signs were monitored throughout treatment and showed no significant abnormalities. This resulted in rapid healing of plaque with mild erythema on the lesional side. After one month follow up, we gave Intralesional Meglumine Antimonate 1-3 ml at the base of the lesion one day apart for 14 days that resulted in compete removal of erythema. We followed up patient after 6 months and it showed complete resolution with no recurrence.

**Figure 1:**
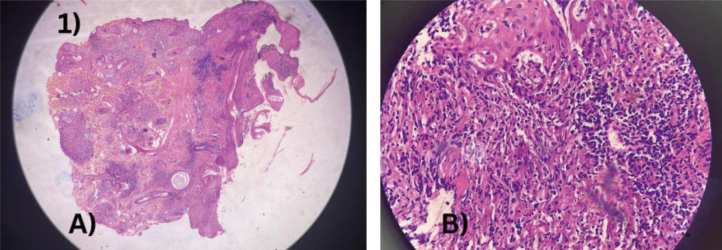
A: Histopathology of the lesion (H&E, × 10) showing epidermal atrophy with dense dermal granulomatous infiltrate composed of epithelioid cells, lymphocytes, and plasma cells. B: Higher magnification (H&E, × 40) demonstrating epithelioid cell granulomas with lymphoplasmacytic infiltrate, consistent with lupoid leishmaniasis.

**Figure 2:**
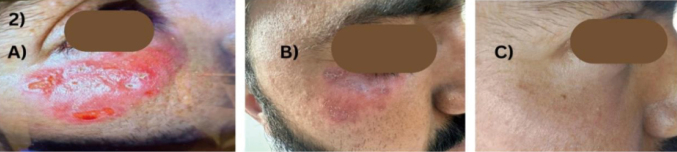
Clinical progression of lesion. A) At initial presentation showing erythematous and infiltrated plaque. B) Regression of lesion after 21 days of Intramuscular injection of Meglumine Antimonate. C) Follow up after 3 months of treatment showing marked improvement.

## Discussion

CL is endemic in Pakistan and its annual burden varies between 50,000 to 100,000 cases^7-10)^. CL is parasitosis carried by bite of phlebotomine sandflies^6)^. There are number of causes like insufficient medical facilities, poverty, minimal planning for vector control, travel of infected persons, female infected sand-flies that influence the spread of disease^11, 12)^.

LCL is a rare form of CL, accounting for 0.5% to 6.2% of cases^13)^. LCL is also known as leishmaniasis recidivans and typically presents as ulcerated, erythematous, infiltrated plaques, nodules, or crusted papules, mainly on exposed areas like the face and limbs^14, 15)^. Differentiating LCL from Lupus Vulgaris (LV) is challenging because both may present with apple jelly nodules, central scarring, and superficial ulceration^16, 17)^. Progressive lesions that are typically present on the face, can be recognized by scar with recurrent marginal papules^18, 19)^. In our case, punch biopsy showed well-formed epithelioid granulomas without caseous necrosis, and Ziehl-Neelsen and PAS stains were negative. Molecular and immunological tests including PCR for Leishmania, GeneXpert, and IGRA were also negative, and the patient had no constitutional symptoms of tuberculosis (weight loss, night sweats, fever). These findings, together with the chronic course, facial location, and positive response to antileishmanial therapy, supported the diagnosis of LCL.

Amastigotes are often absent on parasitological smear in LCL, and cultures may be negative, contributing to delayed diagnosis^17, 20, 21)^. PCR is sensitive in acute CL, but its sensitivity is lower in LCL (≈47%)^16)^. Therefore, clinical features and patient history are crucial for diagnosis.

Pentavalent antimony compounds are currently the primary line of treatment. If there are few lesions, smaller sin size (< 3-4 cm) on accessible locations, then we can give intralesional Meglumine antimonite (MA). Intramuscular injection of MA 20 mg SbV/kg/day for 20-28 days is given in case of multiple lesions (> 5), resistance to local injections, periorificial lesions and proximity to the cartilage/joint^22)^. This is the standard dose of systemic MA in the treatment of CL^23)^. Miltefosine 2.5 mg/kg/day for 28 days is an FDA-approved therapy for CL in individuals over the age of 12^24)^. Many other medications are being utilized as monotherapy in the treatment of CL like paromomycin ointment, cryotherapy, intralesional injections of sodium stibogluconate, pentamidine and oral itraconazole in recent years. LCL is typically resistant to traditional therapies for CL and it can progress slowly for prolonged period^18, 25)^.

## Conclusion

In summary, LCL should be considered in patients with chronic facial plaques and granulomatous histology even when parasitological tests are negative, particularly in endemic regions. Differentiation from LV relies on histopathology, negative tuberculosis testing, molecular assays, and clinical course. Early recognition allows appropriate treatment and prevents unnecessary anti-tuberculous therapy.

## Author contributions

HSB and AK contributed to the conception and design of the case report, clinical management of the patient, and drafting of the manuscript. AH and RS contributed to data collection and review of the clinical records. DSB performed the literature review and assisted in manuscript preparation. HN critically revised the manuscript for important intellectual content and supervised the overall work. All authors read and approved the final version of the manuscript.

## Conflicts of interest statement

The authors declare that there are no conflicts of interest.
